# Potential applications of plant probiotic microorganisms in agriculture and forestry

**DOI:** 10.3934/microbiol.2017.3.629

**Published:** 2017-07-19

**Authors:** Luciana Porto de Souza Vandenberghe, Lina Marcela Blandon Garcia, Cristine Rodrigues, Marcela Cândido Camara, Gilberto Vinícius de Melo Pereira, Juliana de Oliveira, Carlos Ricardo Soccol

**Affiliations:** 1 Bioprocess Engineering and Biotechnology Department, Federal University of Paraná, Curitiba-PR, Brazil; 2 Instituto de Investigaciones Marinas y Costeras (INVEMAR), Santa Marta, Colombia

**Keywords:** plant probiotic microorganisms, plant growth promoters, soil microflora, bioprotectants, biocontrollers, biofertilizers, biostimulants

## Abstract

Agriculture producers, pushed by the need for high productivity, have stimulated the intensive use of pesticides and fertilizers. Unfortunately, negative effects on water, soil, and human and animal health have appeared as a consequence of this indiscriminate practice. Plant probiotic microorganisms (PPM), also known as bioprotectants, biocontrollers, biofertilizers, or biostimulants, are beneficial microorganisms that offer a promising alternative and reduce health and environmental problems. These microorganisms are involved in either a symbiotic or free-living association with plants and act in different ways, sometimes with specific functions, to achieve satisfactory plant development. This review deals with PPM presentation and their description and function in different applications. PPM includes the plant growth promoters (PGP) group, which contain bacteria and fungi that stimulate plant growth through different mechanisms. Soil microflora mediate many biogeochemical processes. The use of plant probiotics as an alternative soil fertilization source has been the focus of several studies; their use in agriculture improves nutrient supply and conserves field management and causes no adverse effects. The species related to organic matter and pollutant biodegradation in soil and abiotic stress tolerance are then presented. As an important way to understand not only the ecological role of PPM and their interaction with plants but also the biotechnological application of these cultures to crop management, two main approaches are elucidated: the culture-dependent approach where the microorganisms contained in the plant material are isolated by culturing and are identified by a combination of phenotypic and molecular methods; and the culture-independent approach where microorganisms are detected without cultivating them, based on extraction and analyses of DNA. These methods combine to give a thorough knowledge of the microbiology of the studied environment.

## 1. Introduction

Plants are never alone, like human beings, they are accompanied by microorganisms, known as plant probiotic microorganisms (PPM), with which they are co-involved in either a symbiotic or free-living association [1]. Human and animal survival has strongly depended on plant kingdom activities, such as agriculture and forestry, throughout history. However, mounting problems associated with world population increase, climate exchange, soil degradation, and environmental contamination have led to the constant need for high yield maintenance.

Because of this, the application of chemical inputs such as pesticides and fertilizers have become a very common practice. Unfortunately, this impacts water and soil pollution, and the whole food chain suffers secondary adverse effects because of this practice, which impacts human health. One strategy to reduce pesticide use has been to select cultivars resistant to a specific disease, but in some cases this resistance can cause damping-off, crown rot, and root rot [1]. Therefore, the use of products based on PPM, known as bioprotectants, biocontrollers, biofertilizers, or biostimulants, is a promising solution to improve the environment quality and ecosystem equilibrium and reduce acquisition costs [2].

This article focuses on the most recent advances concerning PPM as a plant culture improvement and examines some studies and perspectives of modified and improved strain use.

## 2. Plant Probiotic Microorganisms

Today, world population increase, soil degradation, environmental contamination, and climate change affect agriculture and forestry, which are crucial activities for human and animal survival [1]. This has led to plant probiotic microorganism (PPM)-based product development, which is an alternative to biofertilizers, biopesticides, and phytoremediation [3]. Additional new sustainable agriculture concepts, using available environmental resources [4], are being developed.

PPM are beneficial microorganisms that co-evolved with plants in either a symbiotic or free-living association. This association mainly occurs in the soil, but there are other association types, such as microalgae-associated bacteria [5]. Root system soil environments have a high microbial presence due to rhizodeposits and root exudates. Some of these microbes can support their hosts, which stimulates plant growth, reduces pathogen infection, increases yield, and reduces biotic or abiotic plant stresses such as salt stress [3],[5]–[9]. Soil microbial populations consist of plant growth-promoting rhizobacteria, plant disease-suppressive bacteria and fungi, N_2_-fixing cyanobacteria, actinomycetes, and soil toxicant-degrading microbes, among others [4]. However, the growth inhibition in abscisic acid (ABA)-deficient mutant plants by *Bacillus megaterium* [10] is a special case.

In soil, one of the most common groups of PPM is plant growth-promoting bacteria (PGPB), and among these the *Bacilli* and *Pseudomonas* are the most predominant identified genera. The main PGPB source is soil, but some studies have demonstrated the existence of bacteria associated with microalgae that stimulate *Bacillus okhensis* [5] growth. On the other hand, lactic acid bacteria are highly used in fermented and probiotic products and can be considered biocontrollers due to their activity against pathogenic microorganisms and their “generally recognized as safe—GRAS” status, which is included in the FDA's regulations in Title 21 of the Code of Federal Regulations (21 CFR) [11].

Fungi are another highly studied PPM group with important functions. For example, endophytic fungi like *Exophiala* sp. are phytomohormone secretors and can improve plant growth under abiotic stresses [12],[13],[14]. *Trichoderma* strains have also been studied; Palma et al [15] identified molecular mechanisms that are activated during the *in vitro* interaction between tomatoes (*Solanum lycopersicum* L.) and the strain *Trichoderma longibrachiatum* MK1. The results reveal the enrichment of cell defense/stress and primary metabolism categories and promoted changes on secondary metabolism and transport.

**Table 1.** PPM study data and their function.

Microorganism	Strain	Function	Reference
*Alcaligenes faecalis* strain	JBCS1294	Induces plant salt tolerance in *Arabidopsis thaliana*	[7]
*Bacillus subtilis Arthrobacter* sp.	SU47	Alleviates the adverse effects of soil salinity on wheat growth	[17]
*Bacillus megaterium*		Inhibits abscisic acid (ABA) deficient mutant plants	[10]
*Bacillus okhensis*		Promotes early plant growth in *Sorghum bicolor* (L.) Moench	[5]
*Bacillus subtilis*	GB03	Down-regulates expression of the high-affinity K^+^ transporter (HKT_1_)	[9]
*Exophiala* sp.		Promotes growth of a gibberellins (GAs)-deficient mutant cultivar and normal GAs biosynthesis cultivar rice seedlings	[13]
*Fusarium culmorum*	FcRed1	Increases plant biomass and the salt tolerance of rice	[18]
*Halomonas* sp.		Promotes early plant growth	[5]
*Lactobacillus plantarum*	BY	Reduces soft rot disease severity	[11]
*Novosphingobium* sp.		Metabolizes ABA *in vitro*	[19]
*Penicilum minioluteum*		Mitigates the adverse effects of salinity stress in various plants	[14]
*Phoma glomerata Penicillium* sp.	LWL2	Promotes the growth of GAs-deficient dwarf mutant *Waito-C* and Dongjin-beyo rice	[12]
*Pseudomonas fluorescens*		Can help with the enrichment of proteins related to energy metabolism and cell division	[20]
*Sinorhizobium meliloti*		Produces Indole-3-acetic acid (IAA)	[2]
*Trichoderma longibrachiatum*	MK1	Promotes growth and/or increased biotic and abiotic tolerance to stresses	[15]

Unfortunately, PPM performance at field scale is not always satisfactory; the choice of cultivars, the degree and type of fertilization, and crop rotation all influence PPM-plant interactions. However, understanding the distribution and diversity of indigenous bacteria in specific regions, using OMICS technologies, is a promising solution [8],[16]. Table 1 shows PPM study data and PPM role in plant improvement.

## 3. Plant Growth Promoters (PGP)

Plant growth promoters (PGP) are microorganisms: bacteria and fungi that stimulate plant growth through different mechanisms. Some direct PGP action mechanisms include nitrogen amelioration, phosphorous or iron fixation, and plant hormone [21] production. Indirectly, PGP can produce biomolecules as varied as antibiotics, enzymes, and antimicrobial and pathogen-inhibiting volatile compounds, which can lower plant ethylene levels and induce systemic resistance [22],[23],[24]. Rhizospheric and endophytic soil bacteria both present PGP abilities. Rhizosphere microorganisms are typically plant root inhabitants, while endophytic bacteria are either found within the tissues of the plant itself or are free-living soil microorganisms [25].

**Table 2.** PGP strains and their application.

PGP	Effect	Reference
*Trichoderma* spp.	Secondary metabolites produced by *Trichoderma* spp. affected the growth of tomato (*Lycopersicum esculentum*) and canola (*Brassica napus*) seedlings.	[27]
*Trichoderma harzianum*	*T. harzianum* caused effects on maize plant growth but only in combination with mineral fertilization and with disinfected soil as growth substrate.	[28]
*Pseudomonas, Ralstonia, Enterobacter and Pantoea*	Plant growth promotion was evaluated by screening for indoleacetic acid (IAA) production and mineral phosphate solubilization *in vitro.*	[29]
*Bacillus velezensis*	Increased the growth of some tested plants (including beet, carrot, cucumber, pepper, potato, radish, squash, tomato, and turnip) at various levels in different plant parts.	[30]
*Azospirillum* *lipoferum*	Decreased plant water stress in maize (*Zea mays* L.) with abscisic acid (ABA) and gibberellins (GAs) production by *Azospirillum* *lipoferum*.	[31]
Endophytic bacteria (217) and fungi (17) from coffee tissues	Strains were evaluated for their potential to control coffee leaf rust (*Hemileia vastatrix*) and to promote coffee seedling growth. Bacterial strains named 85G (*Escherichia fergusonii*), 161G, 163G, 160G, 150G (*Acinetobacter calcoaceticus*), and 109G (*Salmonella enterica*) increased plant growth. 64R, 137G, 3F (*Brevibacillus choshinensis*), 14F (*S. enterica*), 36F (*Pectobacterium carotovorum*), 109G (*Bacillus megaterium*), 115G (*Microbacterium testaceum*), 116G, and 119G (*Cedecea davisae*) significantly reduced disease severity.	[32]

These PGP have great importance in food production systems, as they increase productivity and reduce the environmental impact caused by agrochemical use [26]. Because of this, several strains have been studied with PGP (Table 2).

### 3.1. Plant nutrition improvement

There are different methods of soil fertilization, such as chemical fertilization, organic fertilization, and microorganism use [33], used to enhance the macro- and micro-nutrients, mainly nitrogen and phosphorous, that favor plant growth and production. Using microorganisms to increase the supply or availability of primary plant nutrients is defined as biofertilization [34]. Nutrient improvement mechanisms include: direct effects on nutrient availability, root growth enhancement, root pathogen antagonists, and soil detritus decomposition [35]. Bacteria and fungi are among the microorganisms that can promote plant growth and have been identified as promoting rhizobateria (PGPR) growth of genera *Acinetobacter*, *Alcaligenes*, *Arthrobacter*, *Azospirillium*, *Azotobacter*, *Bacillus*, *Beijerinckia*, *Burkholderia*, *Enterobacter*, *Erwinia*, *Flavobacterium*, *Pseudomonas*, *Rhizobium*, *Serratia*, and arbuscular mycorrhizal fungi (AMF) [36]–[40].

### 3.2. Plant growth promoting rhizobateria (PGPR)

PGPR greatly contributes to enhanced plant growth and yield [24],[33],[41],[42],[43] because they are part of the rhizosphere biota that, when associated with plants, stimulates host growth. PGPR provides high adaptability in a wide variety of environments, faster growth rate, and a biochemical versatility that allows them to metabolize a wide range of natural and xenobiotic compounds [42],[44].

PGPR can be classified as rhizospheric and endophytic [25],[34] or extracellular and intracellular [45], according to the association between PGPR and the plant host. Extracellular PGPR (ePGPR) exist in the rhizosphere, on the rhizoplane, in the spaces between root cortex cells, and intracellularly (iPGPR) inside root cells, generally in specialized nodular structures [46]. The *Agrobacterium, Arthrobacter, Azotobacter, Azospirillum, Bacillus, Burkholderia, Caulobacter, Chromobacterium, Erwinia, Flavobacterium, Micrococcous, Pseudomonas*, and *Serratia* families are examples of ePGPR [24],[41],[42],[44],[45],[47]. Similarly, *Allorhizobium, Azorhizobium, Bradyrhizobium, Mesorhizobium*, and *Rhizobium*, in the Rhizobiaceae family, are examples of iPGPR. Most rhizobacteria belonging to this group are Gram-negative rods, with a lower proportion being Gram-positive rods, cocci, or pleomorphic [44]. Endophytic or intracellular PGPR generally colonize the specialized nodular root cell structures and include bacteria that fix atmospheric N_2_ [42],[44],[48]. Commercial soybeans (*Glycine max* L.) obtain biologically fixed N through a symbiotic relationship with *Bradyrhizobium japonicum* (Bradyrhizobiaceae) [49].

### 3.3. Arbuscular mycorrhiza fungi

Arbuscular mycorrhiza fungi, or AMF, can enhance the solubility and availability of nutrients, including phosphorous and micronutrients, absorbed by the plant under different conditions [33],[50]. Moreover, AMF affects the phosphorous and nitrogen nutrition of *Medicago sativa* [51] as well as carbon assimilation and water loss through modification of stomatal behavior [52].

The majority of plant roots are symbiotically associated with AMF, mainly the phylum Glomeromycota [53], families Acaulosporaceae (Acaulospora and Entrophospora), Gigasporaceae (Gigaspora and Scutellospora), and Glomaceae (Glomus and Sclerocystis) [54].

The interaction between AMF and bacteria may result in benefic impacts on plants. The presence of bacteria (PGPR) improves fungal colonization in the root, and this interaction positively influences nitrogen and phosphorous enhancement [55],[56]. In addition, phosphate-solubilizing bacterium and AMF improve the availability of carbon and phosphorous compounds [57].

Reforestation enhancement is one important potential AMF application. Since tropical forests are disappearing at the rate of 13.5 million hectares each year, Chaiyasen et al. [58], who investigated the whole of the AMF community in the root structures and rhizosphere soils of *Aquilaria crassna* Pierre ex Lec. and *Tectona grandis* Linn.f. in Thai plantations, set an excellent example. They tried, through fieldwork, to understand whether the AMF communities varied with the host plant species. Terminal restriction fragment length polymorphism, complemented with clone libraries, revealed that AMF community composition in *A. crassna* and *T. grandis* were similar. In this case, a total of 38 distinct terminal restriction fragments (TRFs) were found, 31 of which were shared between *A. crassna* and *T. grandis*. Authors reported that TRFs were attributed to Claroideoglomeraceae, Diversisporaceae, Gigasporaceae, and Glomeraceae.

Regenerating stands of valuable tropical hardwood tree species for sustainable harvest requires the production of seedlings with high probabilities of survival. Chaiyasen et al. [59] stated that AMF employment in root pre-colonization is also a very interesting way to enhance the vigor of plants for outplanting. In the field, the study's authors pursued the strategy of associating the most promising AMF candidates for inoculation with the tree of interest. AMF communities representing four families: *Glomeraceae* (49.6%), *Acaulosporaceae* (24.9%), *Claroideoglomeraceae* (20.8%), and *Gigasporaceae* (4.8%), were assessed in five *Tectona grandis* Linn.f plantations. Each species' plantlets were inoculated *in vitro* and colonized by all studied AMF. Plants inoculated with AMF were taller than non-inoculated plants, which suggests the possibility of using AMF symbiosis in both reforestation and important tree species production in future greenhouse studies.

Other reports cited by Chaiyasen et al. [58] concern AMF's major effects on plant growth, such as nutrient uptake enhancement by plant roots (especially phosphorus) in low-fertility soils [60],[61], plant protection from drought stress [62],[63], plant protection from soil-borne plant pathogenic infection [64], and soil aggregate stability improvement through the mycelia and glomalin action [65],[66],[67].

## 4. Soil Improvement

Sustainable agriculture has increased in recent times, leading to the necessity of new technological developments to reduce environmentally harmful chemical fertilizer and pesticide use. Plant probiotics as an alternative soil fertilization source has been the focus of several studies; their use in agriculture improves nutrient supply and conserves field management and causes no adverse effects [68].

Soil microflora mediates many biogeochemical processes, with some species affecting organic matter and soil pollutant biodegradation (rhizoremediators) and abiotic stress tolerance [68].

### 4.1. Stress tolerance

Abiotic stresses, such as soil salinization, soil solidification, drought, flooding, soil pH, ultraviolet light, heavy metals, and environmental temperature, are major limiting factors in crop production and are the cause of more than 30% of worldwide losses [69]. Salinity and drought, which severely affect plant growth and biomass production, are the two most common abiotic stresses [70].

Under stressful conditions, plants activate a cascade of responses to such situations, which involve primary (changes in ionic or osmotic levels, stomatal closure, and others) and secondary signals (phytohormones and secondary metabolites release). Salinity, nutritional imbalances, and oxidative stress may alter ionic and osmotic plant regulation, while drought affects turgor pressure and biomass production [70],[71],[72].

PGPR interaction with other microorganisms and their effect on the plant physiological response is being studied. However, numerous species of PGPR present an enzyme, known as 1-Aminocyclopropane-1-carboxylic acid (ACC)-deaminase, whose role in the hormonal regulation of plants is already well understood [73]. ACC-deaminase is responsible for the regulation of ethylene, known as the stress hormone; its production in plant roots accelerates in response to both biotic and abiotic processes. When it presents high concentrations it inhibits normal plant development and causes leaf abscission, leaf senescence, chlorosis, and flower wilting, affecting crop yield [4],[74].

Research has shown that plants inoculated with bacteria containing ACC-deaminase show significant increases in high root and biomass due to reduction of ethylene levels, which is beneficial for various stress tolerances [3],[42],[73],[74]. Table 3 shows the benefic effects of PGPR soil application in different cultivars and those cultivars' response to stress tolerance.

Therefore, PGPR, applied as an inoculant in non-favorable soil, may increase microbial diversity, overcoming such problems as abiotic stresses or lack of nutrients and, consequently, improve soil quality, soil health, growth, yield, and crop quality [4].

### 4.2. Bioremediation

As industrial growth has steadily increased, so have environmental pollution levels. Efforts have been made to recover contaminated soil, but conventional hazardous chemical cleanup techniques are both too expensive and harmful to soil microbiota [86]. Some chemical contaminants are petroleum hydrocarbons and polycyclic aromatic hydrocarbons, polychlorinated biphenyls, polychlorinated terphenyls, halogenated compounds like perchloroethylene, and trichloroethylene, as well as pesticides like atrazine and bentazon [87]. Heavy metals, including cadmium, chromium, copper, lead, mercury, nickel, zinc, and others, are the primary inorganic contaminants [88]. Nevertheless, an eco-friendly and low-cost technique has been employed to remediate polluted soil. Phytoremediation involves using plant species capable of accumulating heavy metals for area recovery. The limiting factor for this technique's success is the plant's ability to tolerate large amounts of contaminants without them affecting their biomass [89].

Soil biofertilization with metal-tolerant microorganisms, especially PGPR, has been studied due to their potential for metal bioaccumulation in polluted environments, which enhances metal uptake and promotes plant growth. Microbe-plant association is the main mechanism of bioremediation, which contributes to soil decontamination and plant development [89].

**Table 3.** PGPR application in some cultivars and their biological effects in response to abiotic stress.

Microorganism	Plant	Purpose	Bioinoculation effects	References
Rhizobial strains		ACC deaminase production	ACC deaminase-producing organisms decrease plant ethylene levels that lead to plant growth inhibition or even death	[42]
*Pseudomonas alcaligenes*, *Bacillus polymyxa*, *Mycobacterium phlei*	*Zea mays* (maize)	To investigate the effects of bioinoculants on maize development in two types of soil	The bioinoculant stimulated the plant development and assimilation of N, P, and K in a low nutrient and saline soil	[75]
*Staphylococcus* sp., *Bacillus* sp., *Curtobacterium* sp. M84, *Arthrobacter oxidans* BB1	*Arabidopsis thaliana*	To investigate pathogen resistance and salt stress tolerance	Strains BB1 and M84 showed the best performance under pathogen stress, and BB1 and L81 were better under salt tolerance	[76]
*Pseudomonas mendocina*	*Lactuca sativa* (lettuce)	To investigate the effect of interaction between PGPR and AMF in lettuce under salt stress	*P. mendocina* was able to increase plant biomass under high salt level, while AMF were less effective in alleviating salt stress. Co-inoculation did not show an additive effect on plant growth	[77]
*Pseudomonas* spp.	*Zea mays* (maize)	To evaluate the potential of five *Pseudomonas* spp. in alleviating drought stress in maize	Bioinoculation with *Pseudomonas* spp. improved maize development under drought stress	[78]
*Acetobacter* spp., *Azotobacter* spp., *Rhizobium* spp., *Bradyrhizobium* spp., *Colletotrichum* spp.	*Camellia sinensis* (Tea)	Soil recovery from excessive use of chemical fertilizers	Lower chemical fertilization with biofilm improved soil microbiota and quality, favoring plant growth and rhizoremediation	[79]
*Agrobacterium tumefaciens*, *Zhinguelliuella*, *Brachybacterium* *saurashtrense*, *Vibrio*, *Brevibacterium casei*, *Haererohalobacter*	*Arachis hypogaea*	To investigate the influence of these PRPGs in response of salinity	*A. hypogaea* development decreased in the control treatment under salt conditions. On the other hand, the presence of PGPR promoted plant growth and salt tolerance	[80]
*Azotobacter chroococcum* W5, *Mesorhizobium ciceri* F75, *Pseudomonas striata* P27, *Serratia marcescens* L11 and *Anabaena torulosa*	Wheat seeds	To evaluate the influence of phototroph-heterotroph biofilm on wheat development	Soil treatment with biofilm led to an increase in soil chlorophyll, high concentrations of acetylene reduction activity using *Anabaena Serratia* biofilm, and *Anabaena-Pseudomonas* increased P-uptake	[81]
*Pseudomonas* GGRJ21	*Vigna radiata* (mung bean)	To evaluate how the bacteria promote plant growth and alleviate drought stress	The alleviation of drought stress in mung beans by *Pseudomonas* is related to the production of antioxidant enzymes, cell osmolytes, hormones, and upregulation of stress-responsive genes in the host plant	[82]
PGPR isolated from *Hordeum secalinum* and *Plantago winteri*	*Hordeum vulgare* (Barley)	To isolate rhizobacteria associated with barley and *P. winteri* and evaluate the effect of bioinoculation on barley development under salt stress	The isolate E110 identified as *C. flaccumfaciens* was the most effective growth promoter and stress alleviator	[83]
*Bacillus cereus* Pb25	*Vigna radiate* (mung bean)	To evaluate the influence of bioinoculation in mung bean development under salt stress	*B. cereus* Pb25 showed an important role in mung bean plant protection due to increased production of its plant growth-promoting activity	[84]
*Bacillus amyloliquefaciens* RWL-1	*Oryza sativa* (rice)	To investigate if the abscisic acid producer bacteria is able to protect rice crop from salinity stress	*B. amyloliquefaciens* was able to grow in different salt concentrations without affecting abscisic acid production and produced increased plant height, biomass, and total chlorophyll content	[85]

In a recent study, researchers [90] evaluated PGPR's ability to minimize the arsenic's toxic effect on Malbec grape seedlings. Three species were tested: *Micrococcus luteus*, *Bacillus licheniformis*, and *Pseudomonas fluorescens*, but only *M. luteus* showed a significant arsenic toxic effect decrease and improved biomass growth. In another study, [91] *Bacillus thuringiensis* improved metal accumulation and growth in the species *Alnus firma* in mine tailings soil. Inoculation with *B. thuringiensis* increased root length, shoot height, plant biomass, and heavy metal accumulation in roots and shoots when compared with non-inoculated plants, showing the importance of these consortia in protecting the host plant from environmental stresses and highlighting them as a promising alternative in soil, air, and water recovery.

## 5. Methods to Study the Microbial Ecology of Plants

**Figure 1.**
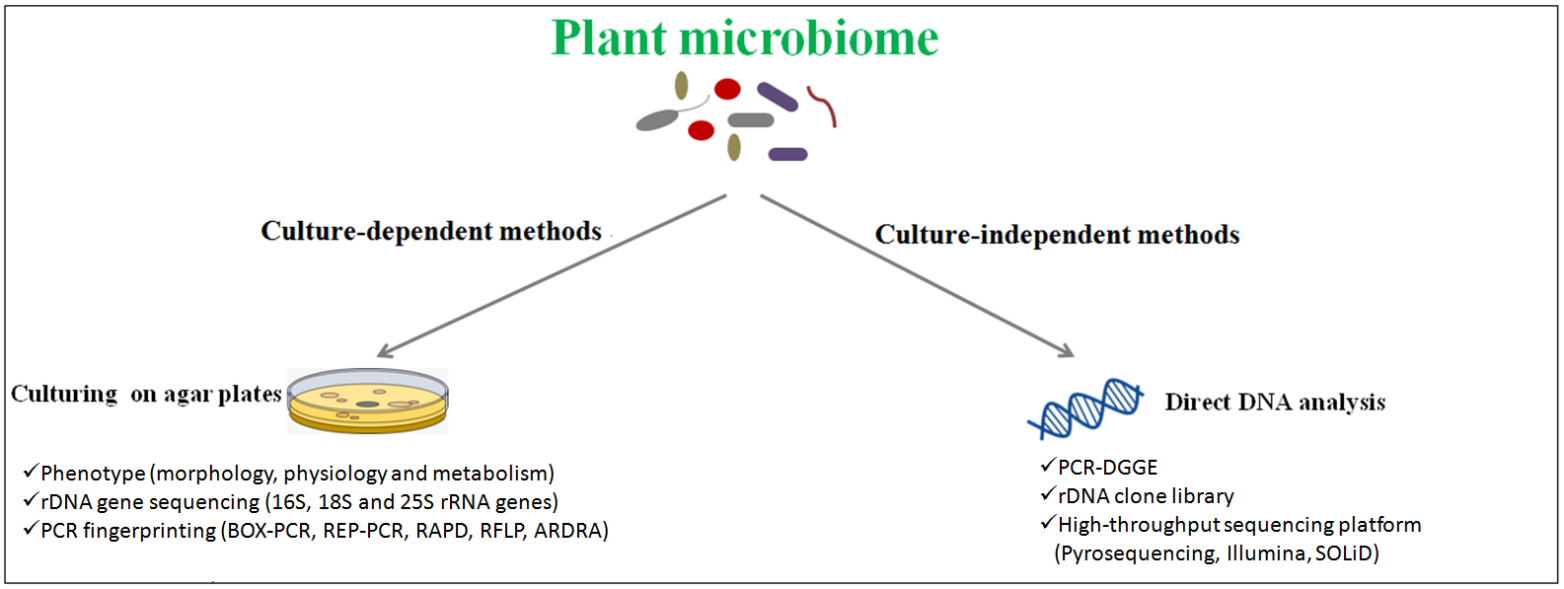
Schematization of methods to access microbial communities of plant materials.

Plant-associated microorganism study is important, not only for understanding their ecological role and their interaction with plants but also to enable the biotechnological application of these cultures to crop management [92]. Current knowledge of microbial community structures in different plant species is based mainly on two different approaches (Figure 1): (1) a culture-dependent approach where microorganisms, isolated from the plant material, are cultivated and identified by a combination of phenotypic and molecular methods; and (2) a culture-independent approach where the microorganisms are detected without cultivation, based on extraction and DNA analyses [93]–[100]. The ideal case combines several approaches for a thorough knowledge of the studied microbiological environment.

### 5.1. Culture-dependent methods

Culture-based tools have long been used to determine the diversity of microorganisms associated with the most diverse plant systems, such as bananas, strawberries, rice, potatoes, maize, eucalyptus, sorghum, soybeans, and wheat [94],[96],[100],[101]. These methods rely on growing microbial cultures in agar plates and then characterizing and identifying them. The most commonly used characteristics for identification are phenotype-related, such as morphological, physiological, and metabolic. As these properties may vary due to both changes in growth conditions and natural mutations, such procedures are insufficient for correct and reliable identification of microorganisms, in addition to being generally time-consuming and laborious. Recent application of newly developed molecular methods to microbial ecology studies overcomes many of these limitations and has led to major advances in understanding plant system microorganism diversity [102].

Sequencing conserved regions of microbial DNA has been broadly used in characterizing plant growth-promoting microorganisms [103]–[106]. In bacterial community investigations, the 16S rRNA gene is normally targeted for PCR amplification, whereas the 26S and 18S rRNA genes are usually targeted in eukaryotic community investigations. Verma et al. [102] applied a polyphasic approach to identify the 217 endophytic bacteria isolated from maize. The combination of biochemical (growth in different carbon sources, intrinsic tolerance to antibiotics, and biochemical tests for catalase, nitrate reductase, and urease), and genetic identification (BOX-PCR and sequencing of the 16S rRNA) revealed the presence of bacteria belonging to the genera *Pantoea*, *Bacillus, Burkholderia*, and *Klebsiella*. A polyphasic approach was evidenced as being important for grouping and identifying this ecosystem's microorganisms [102].

Different methods, such as BOX-PCR, REP-PCR, RAPD, RFLP, AFLP, and ARDRA, of PCR fingerprinting have been used to group and differentiate microbial species in plant materials. All these methods assume that the DNA variations in a microbial strain can be used for classification. Due to the availability of numerous methods in literature, it is sometimes confusing to choose an appropriate method for a particular analysis for two main reasons: (1) microbial culture grouping prior to sequencing; and (2) microbial strain characterization within a species. REP-PCR technique, using the (GTG)^5^ primer, was employed to group bacterial endophytes isolated from strawberry fruit [100]. A total of 45 (GTG)^5^-rep-PCR-clustered isolates were identified by 16S rRNA gene sequencing as *Bacillus subtilis*, *Bacillus* sp., *Enterobacter* sp., *Enterobacter ludwigii*, *Lactobacillus plantarum*, *Pseudomonas* sp., and *Pantoea* sp. The study evidenced the REP-PCR technique's promise as a potential genotypic tool for rapid and reliable clustering of plant bacterial endophytes. Other tools also successfully used to cluster plant microbial cultures were: ADRA-PCR in rice roots [107], BOX-PCR in maize [101], and AFLP in banana roots [108].

### 5.2. Culture-independent methods

Culture-dependent approaches, while extremely useful for understanding isolated microorganisms' physiological potential, do not necessarily provide comprehensive information on microbial community composition [109], because microorganisms can only be cultivated if their metabolic and physiological requirements are previously known [110]. In particular, plants constitute a nutrient-rich ecosystem, and selective media and culture conditions can fail to reproduce the ecological niches and symbiotic relationships behind bacteria–plant and bacteria-bacteria interactions [100]. Culture-independent methods are based on PCR amplification of the microorganisms' rRNA genes; some are as yet uncultivable. This technique has provided valuable insight into community structures, especially those inhabiting nutrient-rich ecosystems, whose growth conditions may be difficult to simulate.

Muyzer et al. [111] set a new milestone by developing a culture-independent method with potential to study the microbial flora quickly and economically, termed denaturing gradient gel electrophoresis (DGGE). This approach has the advantage of directly profiling specific ecosystems' microbial populations by separating PCR products that have originated with universal primers, based on the melting domain of the DNA molecules [112]. PCR-DGGE has shown great potential for aiding the study of different plant species' endophytic bacteria [94],[95],[98],[113]; in fact, a variety of authors have described tremendous differences between isolated and naturally occurring species in such ecosystems. Garbeva et al. [94], using both plating and PCR-DGGE methods, assessed the indigenous bacterial flora diversity associated with potato plants. It was reported that a number of sequences from DGGE analysis (e.g., *Telluria mixta, Caulobacter* sp., *Agromyces cerinus, Afipia genosp.*, and *Pseudomonas* *agarici*) did not have matching sequences from isolates, suggesting that non-culturable or as-yet-uncultured endophytic organisms were detected. On the other hand, Pereira et al. [100] demonstrated that bacteriological culturing of strawberry samples resulted in a more complex microbiota than DGGE analysis. Thus, a combination of conventional culturing methods and PCR-DGGE is needed to understand the ecological role of endophytic bacteria as well as its biotechnological applications in agriculture.

Cloning and sequencing the whole community's rRNA genes is another culture-independent approach used to explore natural ecosystems' microbial diversity. Sequencing of such rRNA gene clone libraries is an appropriate tool with which to gain a more precise picture of a given ecosystem's species diversity. These libraries are being used to reveal the microbial diversity associated with plants [93],[97],[99],[114]. Sun et al. [114] reported a wide diversity of bacteria in rice roots' 16S rDNA library, which consisted of alpha, beta, gamma, delta, and epsilon subclasses of Proteobacteria, Cytophaga/Flexibacter/Bacteroides phyla, low G + C gram-positive bacteria, Deinococcus-Thermus, Acidobacteria, and archaea. In the same way, Sagaram et al. [99], in Dover and Lake Placid in Florida, found a complex bacterial community in citrus groves' 16S rDNA library. A total of 2,062 clones were classified into seven phyla, i.e., Proteobacteria, Bacteroidetes, Dictyoglomi, Actinobacteria, Chlamydiae, Firmicutes, and Verrucomicrobia.

Recently, several massively parallel sequencing platforms, namely next-generation sequencing (NGS), have revolutionized microbial detection by providing greater depth and rare species detection. NGS platforms include technology from 454 Life Sciences (Roche's 454 pyrosequencing) and Solexa's 1G instrument (recently taken over by Illumina and further developed as the Genome Analyzer 2) [115]. A third serious contender is ABI's SOLiD platform. These new technologies perform tens of thousands (or more) sequencing reactions in a single test tube [116].

The new sequencing technologies are making a big impact in plant microbial diversity study [104],[117]. 454 pyrosequencing was used to examine bacterial communities in potato roots [104]. Manter et al. [104] detected 477 bacterial operational taxonomic units, belonging to a total of 238 known genera from 15 phyla, residing within banana roots. Interestingly, five of the ten most common genera (*Rheinheimera, Dyadobacter, Devosia, Pedobacter*, and *Pseudoxanthomonas*) were, for the first time, reported as endophytes of potato plants. Akinsanya et al. [118] used 16S rRNA Illumina sequencing to access endophytic bacteria in *Aloe vera*; the analyses revealed a high and complex bacterial diversity, with a predominance of Proteobacteria, Firmicutes, Actinobacteria and Bacteriodetes. Thus, it is evident that NGS technologies have the power to effectively capture microbial diversity in plant tissues, which can improve our understanding of microbial-plant host interactions [118]. NGS platform use can quickly identify microbial candidates that may be influencing plant growth and production. Finally, meta-transcriptomic analysis could be an additional approach, as it will allow the detection of the genes at work during plant growth.

## 6. Concluding Remarks

Plant probiotic microorganisms (PPM), which are beneficial microorganisms associated with plants, have important function as biofertilizers and biopesticides. The group also contains plant growth promoters (PGP), which are able to stimulate plant growth through different mechanisms including nitrogen, phosphorous or iron fixation, and production of plant hormones. PGP can produce, indirectly, biomolecules as diverse as antibiotics, enzymes, and antimicrobial and pathogen-inhibiting volatile compounds. Molecular characterization and identification of plant-associated microorganisms is very important, not only for understanding their ecological role and plant interactions but also to enable the biotechnological application of these cultures to optimize agriculture productivity.
